# Long Covid, a great imitator of the 21th century

**DOI:** 10.3389/fmed.2022.1026425

**Published:** 2022-09-15

**Authors:** Michel Goldman

**Affiliations:** Institute for Interdisciplinary Innovation in Healthcare (I3h), Université Libre de Bruxelles, Brussels, Belgium

**Keywords:** long Covid, chronic fatigue, fibromyalgia, myalgic encephalomyelitis, COVID-19 vaccine, Epstein Barr virus

## Introduction

As a syndrome which can take multiple forms and mimic different diseases, long Covid can be considered as a novel “Great Imitator,” a nickname attributed to syphilis more than a century ago (1). Indeed, there is a body of evidence that infection with SARS-CoV-2 can result in protean pathological changes which persist after the virus has been cleared, a clinical situation qualified as long Covid or post-COVID-19 condition (2). The impact of long COVID-19 on public health is expected to be huge since more than half of COVID-19 survivors develop post-acute sequelae according to systematic review studies (3–6). The real burden of long Covid is however not established due to the elusive definition of the condition (7) and the emergence of the Omicron variants. Although the risk of developing long Covid after infection with an Omicron variant has been found to be reduced by half as compared to previous strains, the absolute numbers will clearly remain high (8). It is therefore important to rapidly address the several challenges related to long Covid.

## Making the right diagnosis, the clinical challenge

According to the World Health Organization, the diagnosis of long Covid is merely a diagnosis of exclusion that is made in individuals with a history of probable or confirmed SARS CoV-2 infection, who develop long-lasting symptoms that cannot be explained by another cause (2). The list of symptoms that have been attributed to long Covid is very long and most probably not closed. As a matter of fact, post-acute sequelae of COVID-19 may affect almost every system of the human body, with respiratory and neuropsychiatric conditions at the forefront (9, 10). Strikingly, a recent study revealed that the increased risk for seizures, psychosis, dementia and epilepsy persists for at least 2 years (11).

In view of the wide range of symptoms and the lack of biomarkers, the diagnosis of long Covid requires experienced physicians with broad clinical knowledge. The first step in the work-up of a patient suspect to suffer from this condition is to carefully look at his/her past medical history before he/she was infected with the SARS-CoV-2 virus. Indeed, it is not unusual to identify somatic symptoms which have occurred before the acute COVID-19 phase (12). In such cases, one can assume that these symptoms were not primary caused but rather exacerbated by the infection. The next step is to get a precise description of the acute phase of the infection. This might allow to distinguish lingering symptoms following the acute disease from new symptoms that appear after resolution of the acute disease (9). In most cases, appropriate clinico-biological and imaging explorations will be needed to exclude diagnoses unrelated to COVID-19.

When cognitive dysfunction, depression, fatigue associated with musculoskeletal pain, post-exertional malaise, sleep disturbances or orthostatic intolerance are present, long Covid might be confounded with myalgic encephalomyelitis, chronic fatigue or fibromyalgia (13). Although the pathophysiology of these conditions is unclear, there is evidence that viral infections might represent contributing factors. Indeed, they might develop after infectious mononucleosis/ glandular fever caused by the Epstein Barr virus as well as after the Severe Acute Respiratory Syndrome (SARS) and the Middle East Respiratory Syndrome (MERS) caused by β-coronaviruses closely related to SARS-CoV-2 (14). Long Covid can be considered as a post-viral syndrome of a similar type.

## Deciphering the pathophysiological determinants, the research challenge

Whereas, the immunoinflammatory processes elicited by the SARS-CoV-2 virus in the acute phase of COVID-19 have been reasonably well characterized (15), the pathophysiology of long Covid remains obscure although a number hypothetical mechanisms have been proposed (16, 17). Viral persistence might be an important factor as suggested by a study demonstrating persistence of viral RNA and viral antigens in the gut mucosa of patients with long Covid (18). Despite experimental evidence that the SARS-CoV-2 virus might enter the central nervous system (CNS), there so far no demonstration that neuro-psychiatric symptoms of long Covid are caused by infection of brain cells (19).

Incomplete healing of early inflammatory or thrombotic lesions elicited by the virus might certainly explain several persistent symptoms, especially in the lungs (9). There is indeed suggestive evidence that early events increase the risk to develop long Covid, even in patients who were not hospitalized (20–22). Among these events, reactivation of the Epstein Barr virus deserves special attention (22).

Immune dysregulation could also play an important role in the pathogenesis of long Covid, First and foremost, a diverse range of autoantibodies were detected in the serum of COVID-19 patients (23). Anti-nuclear antibodies and antibodies neutralizing interferon-α might be especially important to consider as their production was found to correlate with the development of long Covid features (22). Besides humoral autoimmunity, there is suggestive evidence that T lymphocytes either virus-specific or activated by bystander mechanisms could also contribute to the pathology of long Covid (22, 24).

## Taking care of long Covid patients, the therapeutic challenge

Given the heterogeneity of long Covid and the poor knowledge on its pathogenesis, it is not surprising that its treatment is merely symptomatic. Pain medications including opioids, antidepressant drugs and anxiolytic drugs are frequently used (10), often without adequate surveillance. Although guidelines have been proposed by several learned societies, certain therapeutic modalities remain a matter of debate such as behavioral therapy and grade exercise therapy for symptoms suggestive of myalgic encephalomyelitis or chronic fatigue (17).

Since viral persistence might contribute to long Covid, it is important to delineate the impact of anti-SARS-CoV-2 vaccination and antiviral drugs in patients suffering from post-Covid conditions. There is evidence that vaccination might reduce symptoms in some patients suffering from long Covid but these beneficial effects are limited (25, 26). Likewise, when acute Covid occurs despite vaccination, the risk of developing long Covid is only moderately reduced after breakthrough infection (27–29). Regarding antiviral drugs, the observation of viral rebounds after a 5-day course of nirmatrelvir-ritonavir (30) raises significant concerns about the effect of this treatment on long Covid. Undoubtedly, additional research is needed to efficiently tackle long Covid. In the meantime, it is important to make healthcare professionals and patients savvy about the several uncertainties that we are facing.

## Concluding remarks

Long covid is a highly heterogeneous condition with multiple clinical presentations of variable severity and multiple factors contributing to its pathogenesis. There is an urgent need to foster new interdisciplinary research to stratify long Covid patients according to phenotypic and molecular criteria with the ultimate goal to provide each of them with the right treatment at the right time.

## Author contributions

The author confirms being the sole contributor of this work and has approved it for publication.

## Funding

This work was supported by a grant of the Université libre de Bruxelles to the I3h Institute.

## Conflict of interest

The author declares that the research was conducted in the absence of any commercial or financial relationships that could be construed as a potential conflict of interest.

## Publisher's note

All claims expressed in this article are solely those of the authors and do not necessarily represent those of their affiliated organizations, or those of the publisher, the editors and the reviewers. Any product that may be evaluated in this article, or claim that may be made by its manufacturer, is not guaranteed or endorsed by the publisher.
